# Lipid nanoparticles based on natural matrices with activity against multidrug resistant bacterial species

**DOI:** 10.3389/fcimb.2023.1328519

**Published:** 2024-01-09

**Authors:** Elenice Francisco da Silva, Luciana Machado Bastos, Belchiolina Beatriz Fonseca, Rosineide Marques Ribas, Simone Sommerfeld, Henrique Machado Pires, Fernanda Aparecida Longato dos Santos, Lígia Nunes de Morais Ribeiro

**Affiliations:** ^1^ Institute of Biotechnology, Federal University of Uberlandia, Uberlandia, Brazil; ^2^ School of Veterinary Medicine, Federal University of Uberlandia, Uberlandia, Brazil; ^3^ Institute of Biomedical Sciences, Federal University of Uberlandia, Uberlandia, Brazil

**Keywords:** multi-resistant bacteria, nanostructured lipid carriers, vegetable butter, essential oil, natural antibiotics

## Abstract

Lately, the bacterial multidrug resistance has been a reason to public health concerning around world. The development of new pharmacology therapies against infections caused by multidrug-resistant bacteria is urgent. In this work, we developed 10 NLC formulations composed of essential oils (EO), vegetable butter and surfactant. The formulations were evaluated for long-term and thermal cycling stability studies in terms of (particle size, polydispersion index and Zeta potential). *In vitro* antimicrobial assays were performed using disk diffusion test and by the determination of the minimum inhibitory concentration (MIC) performed with fresh and a year-old NLC. The most promising system and its excipients were structurally characterized through experimental methodologies (FTIR-ATR, DSC and FE-SEM). Finally, this same formulation was studied through nanotoxicity assays on the chicken embryo model, analyzing different parameters, as viability and weight changes of embryos and annexes. All the developed formulations presented long-term physicochemical and thermal stability. The formulation based on cinnamon EO presented *in vitro* activity against strains of *Acinetobacter baumannii*, *Klebsiella pneumoniae* and *Pseudomonas aeruginosa* isolated from humans and *in vivo* biocompatibility. Considering these promising results, such system is able to be further tested on *in vivo* efficacy assays.

## 1 Introduction

The emergence of multi-resistant bacteria (MDR) to antibiotics represents one of the principal global threats to public health (Murray et al., 2022). The clinicians have been considered the use of the last therapeutics options, such as vancomycin, polymyxin B, carbapenems and colistin, with low rates of success (Wang et al., 2022; Yao et al., 2023). The infections by MDR are currently associated with considerable increase in morbidity and mortality of intensive care units (Braga et al., 2018), increasing hospital costs (Osme et al., 2020) and resistance against the strategies used to combating them, as antibiotics association (Batchelder et al., 2023). There is an estimative that by 2050 will there will be 10 million deaths from MDR, annually (O’Neill, 2016).

In addition, in the COVID-19 pandemic, there was an indiscriminate use of broad-spectrum antibiotics (Freire et al., 2023), increasing the antimicrobial resistance in worldwide (Kariyawasam et al., 2022), as reported for bacterial resistance to carbapenems (Pan American Health Organization, 2021).

In this sense, the use of compounds of natural origin, such as essential oils (EO) can represent an interesting alternative to combat infections by MDR pathogens. EO are promising drug candidates, once they have several intrinsic biological properties, such as antibacterial, antifungal, antiviral, antioxidant, anticancer, analgesic, anti-inflammatory, biopesticide, repellent, among others (Elshafie and Camele, 2017; Cimino et al., 2021). These compounds come from the secondary metabolism of plants, being extracted from bark, leaves, flowers, buds, seeds, roots and fruits. Its chemical constitution is complex, being mainly composed of monoterpenes, sesquiterpenes and terpenoids (El Asbahani et al., 2015). However, the application of EO in the clinical approaches is limited, due to its low solubility in water, low physicochemical stability, high volatility and hydrolysis (Carbone et al., 2018; Guidotti-Takeuchi et al., 2022). Moreover, the toxicity of the esophageal and gastrointestinal mucosa limits its use by enteral administration route, as well as allergic reactions can be observed in the skin if they are insufficiently diluted (Lammari et al., 2021).

Thus, the EO nanoencapsulation can be an interesting alternative to overcome such limitations. The lipid nanoparticles, including the nanostructured lipid carriers (NLC), which is composed by solid and liquid lipids at room temperature (Ribeiro et al., 2021), seems to be an excellent strategy. NLC are biodegradable, biocompatible, scalable and ecofriendly (Dupuis et al., 2022). Such systems allow the encapsulation of hydrophobic molecules as EO, improving its physicochemical stability (Cimino et al., 2021), protecting against degradation and evaporation. This process still increases the EO solubility in water, optimizing its bioavailability, allowing its sustained release (Katopodi and Detsi, 2021) and, consequently, decrease the EO intrinsic toxicity (Dajic Stevanovic et al., 2020).

This work reports the development of NLC formulations based on natural matrices composed of vegetable butter and EO with activity against strains of *Acinetobacter baumannii*, *Klebsiella pneumoniae* and *Pseudomonas aeruginosa* isolated from humans. These bacterial species were selected because they are drug multi-resistant, including the resistance to antibiotics considered the last therapeutic choice. Consequently, they are the most prevalent species of the most of infections described in clinics, increasing hospital costs and mortality rates (Tacconelli et al., 2018; Osme et al., 2020; World Health Organization, 2021; Murray et al., 2022; Wang et al., 2022; Micheli et al., 2023; Yao et al., 2023). Firstly, a screening of 14 natural oils was carried out to select those with the most promising *in vitro* antibacterial activity as the structural and bioactive excipient in the preparation of different NLC formulations. The resultant formulations were monitored by a long-term physicochemical stability study (1 year, 25°C), followed by thermal cycling stability test. Then, the *in vitro* antibacterial activity was evaluated through the determination of the minimum inhibitory concentration (MIC). Finally, the structural characterization and the *in vivo* nanotoxicity test on chicken embryo model (CE) were performed to determinate compatibility between the excipients, thermal stability and the safety of the most promising nanosystems.

## 2 Materials and methods

### 2.1 Materials

Ucuuba butter, cinnamon bark EO (*Cynnamomum zeylanicum*), oregano EO (*Origanum vulgare*), tea tree EO (*Melaleuca artenifolia*), peppermint EO (*Mentha pipperita*), rosemary EO (*Rosmarinus officinalis*), clove EO (*Eugenia caryophyllus*), ginger EO (*Zingiber officinale*), salvia EO (*Salvia sclarea*), basil EO (*Ocimum basilicum*), citronella EO (*Cymbopogon nardus*), star anise EO (*Illicium verum*), frankincense EO (*Boswellia carterii*), candeia (*Eremanthus erythropappus*) and copaiba (*Copaifera officinalis*) vegetable oils were commercially obtained from the company Engetec/Engenharia das Essências^®^, Brazil. Poloxamer (Kolliphor^®^ P188), used as a surfactant, was purchased from Sigma-Aldrich, USA. Deionized water (18 MΩ) was obtained from a Milli-Q^®^ Direct 8 ultra-pure water purifier. Bacterial isolates of *A. baumannii* (65JF, OXA-23 and POA4-28), *K. pneumoniae* (ATCC700603, Responsive and Bactray) and *P. aeruginosa* (IMP-1 and PAO-1) were obtained from the collection of the Molecular Microbiology Laboratory of the Federal University of Uberlandia, Brazil. *Klebsiella pneumoniae* ATCC700603 is a standard strain. Responsive *K. pneumoniae* was isolated from a hospitalized patient in Uberlandia – MG that we used as a secondary control for sensitivity to antibiotics. All the other strains were isolated from hospitalized patients in Brazil and characterized as MDR, once they came from an acquired resistant infection not susceptible to at least 1 agent in 3 or more antimicrobial categories (Magiorakos et al., 2012). The medium culture used were: Soy Triptone Agar (Isofar, Brazil), MacConkey Agar (Ion, India), Mueller Hinton Agar (Laboratorios Conda S.A., Spain), Mueller Hinton Broth (Biolog^®^). Disks impregnated with Polymyxin B were purchased from Cefar Diagnóstica Ltda, São Paulo, Brazil.

### 2.2 Screening of vegetable and essential oils

The screening of different vegetable and EO was carried out through the disk diffusion test against *A. baumannii*, *K. pneumoniae* and *P. aeruginosa* strains. Commercial polymyxin B disks were used as a positive control. The strains of interest were inoculated in Soy Triptone agar (TSA) or MacConkey agar medium culture, separately, and incubated at 37°C for 24 h. After this period, isolated colonies of 3 to 5 species of the same morphological type were collected and diluted in 10 mL of 0.9% sterile saline solution, with turbidity adjusted according 0.5 MacFarland scale, up to a final concentration of colony forming units 1.5 x 10^8^ (CFU) mL^-1^ (Ribeiro et al., 2021). The resulting bacterial suspension was used in the disk diffusion test (pure EO, screening stage) and in the MIC determination assay. Plates containing Mueller Hinton agar were previously prepared. With a sterile swab, each bacterial suspension analyzed (*A. baumannii*, *P. aeruginosa* and *K. pneumoniae*) was equally distributed over the agar surface and stored by 5 min (25°C). After this period, disks impregnated with polymyxin B (positive control) and EO were positioned over the agar, in duplicate. Approximately 20 μL of each pure oil was added to sterile filter disks (6 mm in diameter) immediately before being positioned on the plates. The plates were incubated at 37 ± 1 °C for 24 h (Ribeiro et al., 2020). Finally, the diameters growth inhibition zones (mm) were registered using a millimeter scale. One-way ANOVA/Tukey *post hoc* statistical tests were used to evaluate intergroup statistical differences (p< 0.05), determined by Microsoft Excel Professional Plus 2019 and Action version 2.9 software.

### 2.3 Preparation of nanostructured lipid carriers

Different NLC formulations were prepared by the hot ultrasonication-emulsification method. The lipid phase of NLC was composed of ucuuba butter (solid lipid) and EO (liquid lipid) with a total lipid concentration of 10% (w/v) at a ratio of liquid lipids to total lipids of 0.1:1. So, the final EO concentration in NLC was 10 mg/mL, corresponding to 10% of the total lipid matrix of NLC. Briefly, in a water bath, the lipid phase was heated 10°C above the melting point of the solid lipid (ucuuba butter). Simultaneously, the aqueous phase containing 5% poloxamer was heated to the same temperature as the lipid phase. Then, the aqueous phase was added dropwise to the lipid phase, under stirring at 10,000 rpm for 2 min, in an Ultra-Turrax high pressure homogenizer (Ultra-Turrax^®^ T18). The obtained microemulsion was immediately ultrasonicated for 20 min in a Vibracell tip sonicator (Sonics & Mat. Inc., Danbury, USA) operated at 500 W and 20 kHz, with alternating cycles (on/off) at 30 s. After this step, the formed nanoemulsion was immediately cooled to room temperature (25° C) in an ice bath to solidify the nanoparticles (Ribeiro et al., 2021). For control experiments, solid lipid nanoparticles (without EO addition) were prepared following the same procedure. For didactic purposes, this system was called NLC control in this work (**Table 1**).

**Table 1:** Composition and concentration of total lipids (TL), ucuuba butter (LS), essential oils (EO) and surfactant used in each NLC formulation.

NLC	TL (%)	LS (%)	EO (%)
**F1**	**10**	**9**	**Cinnamon, 1%**
**F2**	**10**	**9**	**Oregano, 1%**
**F3**	**10**	**9**	**Pepper mint, 1%**
**F4**	**10**	**9**	**Tea tree, 1%**
**F5**	**10**	**9**	**-**
**F6**	**10**	**9**	**Cinnamon and Oregano, 1%**
**F7**	**10**	**9**	**Cinnamon and Pepper mint, 1%**
**F8**	**10**	**9**	**Cinnamon and Tea tree, 1%**
**F9**	**10**	**9**	**Oregano e Pepper mint, 1%**
**F10**	**10**	**9**	**Oregano e Tea tree, 1%**
**F11**	**10**	**9**	**Tea tree e Pepper mint, 1%**

TL = LS + OE; All NLC were stabilized with poloxamer 5% (w/v).

### 2.4 Long-term physicochemical stability study

The nanoparticle size (nm), polydispersion index (PDI) and Zeta potential (mV) were analyzed by diluting the NLC formulations (1:1000 v/v) in deionized water through the dynamic light scattering (DLS) technique by Litesizer equipment (Anton Paar Instruments, Anton Paar, Berlin, Germany). The same parameters were monitored for 12 months at 25°C, in triplicate (Ribeiro et al., 2017). One-way ANOVA/Tukey *post hoc* statistical tests were used to evaluate intragroup statistical differences over time (p< 0.05), calculated by GraphPad Prism 8 software.

### 2.5 Evaluation of *in vitro* antibacterial activity

All prepared NLC were evaluated by microdilution method to determine the MIC. This experiment was performed in 96-well plates, in triplicate. Different strains of *A. baumannii*, *P. aeruginosa* and *K. pneumoniae* isolated from humans were analyzed. Bacterial suspensions were prepared as previously detailed in the screening step. They were diluted in 96-well plate to a final concentration per well of 1 × 10^6^ CFU·mL^-1^. Dilutions of all formulations were added into 96-well plates to reach 0.1 mL final volume. The negative control was Mueller Hinton Broth without bacteria. The 96-well plate of each bacterial strain was incubated at 37°C for 24 h. After this period, the MIC of *A. baumannii*, *P. aeruginosa* and *K. pneumoniae* was determined for each NLC. One-way ANOVA/Tukey *post hoc* statistical tests were adopted to evaluate inter-group statistical differences (p< 0.05), determined by Microsoft Excel Professional Plus 2019 and Action version 2.9 software.

### 2.6 Structural characterization

The structural characterization was performed by infrared spectroscopy measurement (FTIR-ATR), differential scanning calorimetry (DSC) and field emission scanning electron analyses (FE-SEM). In the FTIR-ATR technique, the spectral range was 650 to 4000 cm^-1^, with resolution of 2 cm^-1^. The DSC analyses were performed using a TA equipment, model Q20. Samples were added in sealed aluminum pans. Analyzes were carried out in a nitrogen atmosphere, at a flow rate of 50 mL/min^-1^, in the temperature range from 0 to 100°C, at a heating rate of 10°C/min. In order to elucidate the morphology of NLC, FE-SEM analyses was performed using Tescan brand, model VEGA 3 microscope, operating in high vacuum under a voltage of 20 kV. Briefly, a drop of each sample was adhered to a cover glass previously fixed in a stub. After, the stubs were subjected to sputtering procedure for 120 s at 30 kV. Then, the samples were visualized in different magnifications.

### 2.7 Thermal cycling stability study

The thermal stability was evaluated by 3 consecutive temperature cycles totaling 21 days of monitoring in a Climatic Chamber Stability (Solab, Piracicaba, Brazil). Each cycle corresponded to 7 days of NLC storage under different critical conditions, such as: 4 days at 40°C followed by 3 days at 15°C. At the end each cycle, nanoparticle size (nm), polydispersity index (PDI) and Zeta potential (mV) were measured, by DLS through Litesizer equipment (Anton Paar Instruments, Anton Paar, Berlin, Germany), in triplicate. One-way ANOVA/Tukey *post hoc* statistical tests were used to evaluate intragroup statistical differences over time (p < 0.05), calculated by GraphPad Prism 8 software.

### 2.8 *In vivo* nanotoxicity assay on chicken embryo model

The nanotoxicity of NLC formulation was evaluated through *in vivo* chicken embryo model (CE), according to the following parameters: viability (%) and weight (g) (Ribeiro et al., 2020). 80 eggs of *Gallus gallus*, lineage W-36, were used. Before analyses, the eggs were submitted to ovoscopy to ensure the viability of the embryos at 7 days of development. Then, they were weighted and divided into 10 CE groups (n = 8) as follow: negative control (NC), eggs treated with 0.85% saline; NLC control (without EO), cinnamon EO emulsified with poloxamer (both at the same concentration as the NLC formulation) and NLC/CAN. All treatments were administered on CAM of CE at different concentrations: 0.5, 0.3 and 0.1% (w/v). Then, the eggs were incubated in automatic incubator (Premium Ecológica^®^) at 37.5°C and 55% air humidity for 7 days. The CE mortality was daily observed to determine the viability (%). At 14 days of embryonic development, the eggs were weighted.

The weight changes of CE were calculated through the difference between the weight of the eggs before and after the treatments, according to the following equation:


aW=(ce.ysW × 50)÷ieW

where aW = egg weight adjusted to 50 g; ce.ysW = embryo or annexes weight on 14 day of embryonic develop; ieW = initial egg weight on 7 day of embryonic develop.

The One-way ANOVA/Tukey tests were used to elucidate the intergroup statistical differences, in terms of CE weight (p< 0.05). For the CE viability test, chi-square test was used followed by the difference between two proportions test (p< 0.05). GraphPad Prism 8 software was used for these analyses.

## 3 Results

### 3.1 Screening of vegetable and essential oils

A preliminary screening of 14 natural oils was carried out in order to select those with the best activity against those microbial strains, in comparison with polymyxin B (**Table 2**). It was observed that cinnamon and oregano EO have inhibited all bacterial species and strains tested. Cinnamon EO exhibited inhibition zones ranging from 21.0 to 37.5 mm while oregano EO was responsible for inhibition zones ranging from 9.5 to 39.5 mm. Polymyxin B exhibited an average inhibition zone of 14.1 mm, considering all strains of the bacterial species evaluated. The assay also showed that peppermint EO and tea tree EO were capable of inhibiting the bacterial growth, producing inhibition zones ranging from 10.0 to 33.0 mm and 10.5 to 20.0 mm, respectively. Then, these EO were nanoencapsulated as active and structural compound of NLC.

**Table 2:** Growth inhibition zone of different bacterial species treated with different EO.

MEAN DIAMETER OF BACTERIAL GROWTH INHIBITION ZONE (mm)								
**EO**	*A. baumannii* 65JF	*A. baumannii* OXA-23	*A. baumannii* POA4-28	*K. pneumoniae* ATCC700603	*K. pneumoniae* Bactray	*K. pneumoniae* Responsive	*P. aeruginosa* IMP-1	*P. aeruginosa* PAO-1
Rosemary	0.00±0.00	0.00±0.00	9.00±1.41	0.00±0.00	0.00±0.00	0.00±0.00	0.00±0.00	2.00±0.00
Star anise	9.50±0.70	0.00±0.00	14.00±0.00***	0.00±0.00	0.00±0.00	0.00±0.00	0.00±0.00	0.00±0.00
Basil	14.50±2.12***	12.50±0.70***	24.00±1.41*	4.50±0.70	13.50±0.70***	9.00±0.00***	11.00±1.41**	0.00±0.00
Cinnamon	30.00±0.00*	37.50±3.53*	31.50±0.70*	30.50±0.70*	40.00±1.41*	31.00±1.41*	34.50±6.36*	21.00±1.41*
Citronella	10.00±0.00	0.00±0.00	13.00±1.41***	0.00±0.00	18.00±2.82***	0.00±0.00	18.50±2.12 **	0.00±0.00
Clove	12.50±2.12***	11.50±0.70***	19.00±1.41**	9.50±0.70	14.00±1.41***	15.00±0.00**	15.00±0.00**	0.00±0.00
Peppermint	13.50±0.70***	0.00±0.00	25.00±7.07*	0.00±0.00	26.00±1.41**	10.00±1.41***	33.00±4.24*	0.00±0.00
Tea tree	15.00±0.00***	14.00±2.82***	20.00±11.31**	10.50±0.70	13.00±1.41	13.00±0.00**	16.00±0.00**	0.00±0.00
Oregano	22.50±0.70**	26.50±4.94**	35.50±6.36*	20.50±0.70**	39.50±0.70*	30.00±0.00*	30.00±0.00*	9.50±0.70**
Salvia	0.00±0.00	0.00±0.00	0.00±0.00	0.00±0.00	17.00±1.41***	0.00±0.00	9.50±0.70***	6.00±1.41***
Polymyxin B	15.00±0.00***	12.50±2.12***	13.00±0.00***	15.00±0.00***	13.50±2.12***	15.00±0.00**	14.00±0.00**	15.00±0.00*

NOTE: Means in the same column followed by distinct asterisks differ statistically from each other p < 0.05 (One way ANOVA followed by Tukey test);

* Represents the highest values of means similar to each other;

** Represents the second highest values of similar means;

*** Represents the third highest values of similar means.

### 3.2 Long-term physicochemical stability study

**Figure 1:**
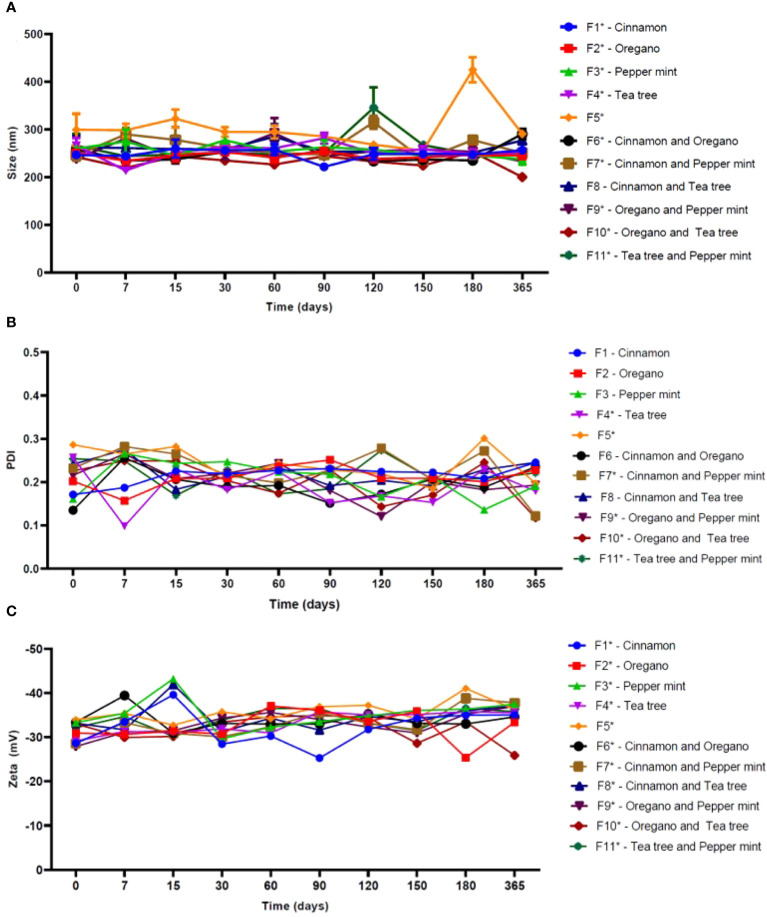
Physicochemical stability of NLC formulations in terms of size **(A)**, PDI **(B)** and Zeta potential **(C)**, over 365 days at 25°C (n = 3). NOTE: One-way ANOVA/Tukey were used to analyze intragroup statistics; *p< 0.05. PDI: polydispersion index.

**Figure 1** showed the results of the long-term physicochemical stability monitoring of the investigated formulations. The control formulation (F5) exhibited an average particle size of 303.5 ± 46.3 nm statistically higher (p< 0.05) than the others NLC based-EO, with the highest fluctuation in particle size over time.

Among the samples, only F8 formulation showed no statistically significant changes (p = 0.09) over time. Mean values ​​of PDI ranged from 0.11 to 0.23 among the systems containing EO in its structure, while NLC control had a mean value of 0.24. In this case, F1 (p = 0.36), F2 (p = 0.45), F3 (p = 0.08), F6 (p = 0.05) and F8 (p = 0.22) did not show significant changes over time. The average values of Zeta potential of F5 were -35.7 mV. Among the systems containing EO, these values ​​ranged from -32.2 to -35.2 mV. In this parameter, all NLC showed statistically significant variations (p< 0.05) over time.

### 3.3 Evaluation of the *in vitro* antibacterial activity

All prepared NLC were submitted to the *in vitro* antimicrobial activity test against the different bacterial strains through MIC test. This assay determined that the formulations F1, F6, F7 and F8 showed antimicrobial activity against all bacterial species and strains evaluated. However, F1 formulation, composed of cinnamon EO, ucuuba butter and stabilized by poloxamer, was the most efficient system against *A. baumannii*, *P. aeruginosa* and *K. pneumoniae* inhibiting their growth, in the lowest possible concentration (**Table 3**). Thus, F1 after 12 months of storage was submitted to MIC assay again, in order to elucidate if the antimicrobial activity was maintained over time. It was observed that this nanosystem maintained the activity against all bacterial species investigated. Moreover, MIC values after a year was higher than that observed at the beginning of the study (**Table 3**) with the exception of Bactray *K. pneumoniae*, Responsive *K. pneumoniae* and IMP-1 *P. aeruginosa* species.

**Table 3:** Minimum inhibitory concentration (mg/mL^-1^) of NLC against different bacterial strains.

	Treatment mg/mL^-1^							
Species	After 2 months							After 12 months
strains	F1	F2	F6	F7	F8	F9	F10	F1
*A. baumannii*								
OXA-23	1.19 ± 0.00	> 5.00	3.33 ± 2.89	1.60 ± 2.78	3.33 ± 1.44	> 4.90	> 5.00	> 4.76
POA4-28	0.80 ± 0.34	1.25 ± 0.00	1.25 ± 0.00	1.20 ± 0.00	1.25 ± 0.00	3.27 ± 2.83	2.08 ± 0.72	1.19 ± 0.00
65JF	1.19 ± 0.00	> 5.00	5.00 ± 0.00	3.20 ± 1.39	2.50 ± 0.00	> 4.90	> 5.00	2.38 ± 0.00
*K. pneumoniae*								
ATCC700603	1.19 ± 0.00	> 5.00	5.00 ± 0.00	4.81 ± 0.00	5.00 ± 0.00	> 4.90	> 5.00	4.76 ± 0.00
Bactray	1.19 ± 0.00	> 5.00	2.50 ± 0.00	2.40 ± 0.00	2.50 ± 0.00	> 4.90	> 5.00	1.19 ± 0.00
Responsive	1.19 ± 0.00	> 5.00	2.50 ± 0.00	2.40 ± 2.41	2.50 ± 0.00	> 4.90	> 5.00	1.19 ± 0.00
*P. aeruginosa*								
IMP-1	1.19 ± 0.00	> 5.00	2.50 ± 0.00	2.40 ± 0.00	2.50 ± 0.00	> 4.90	> 5.00	1.19 ± 0.00
PAO-1	1.19 ± 0.00	> 5.00	5.00 ± 0.00	4.81 ± 0.00	4.17 ± 1.44	> 4.90	> 5.00	2.38 ± 0.00

### 3.4 Structural characterization

**Figure 2:**
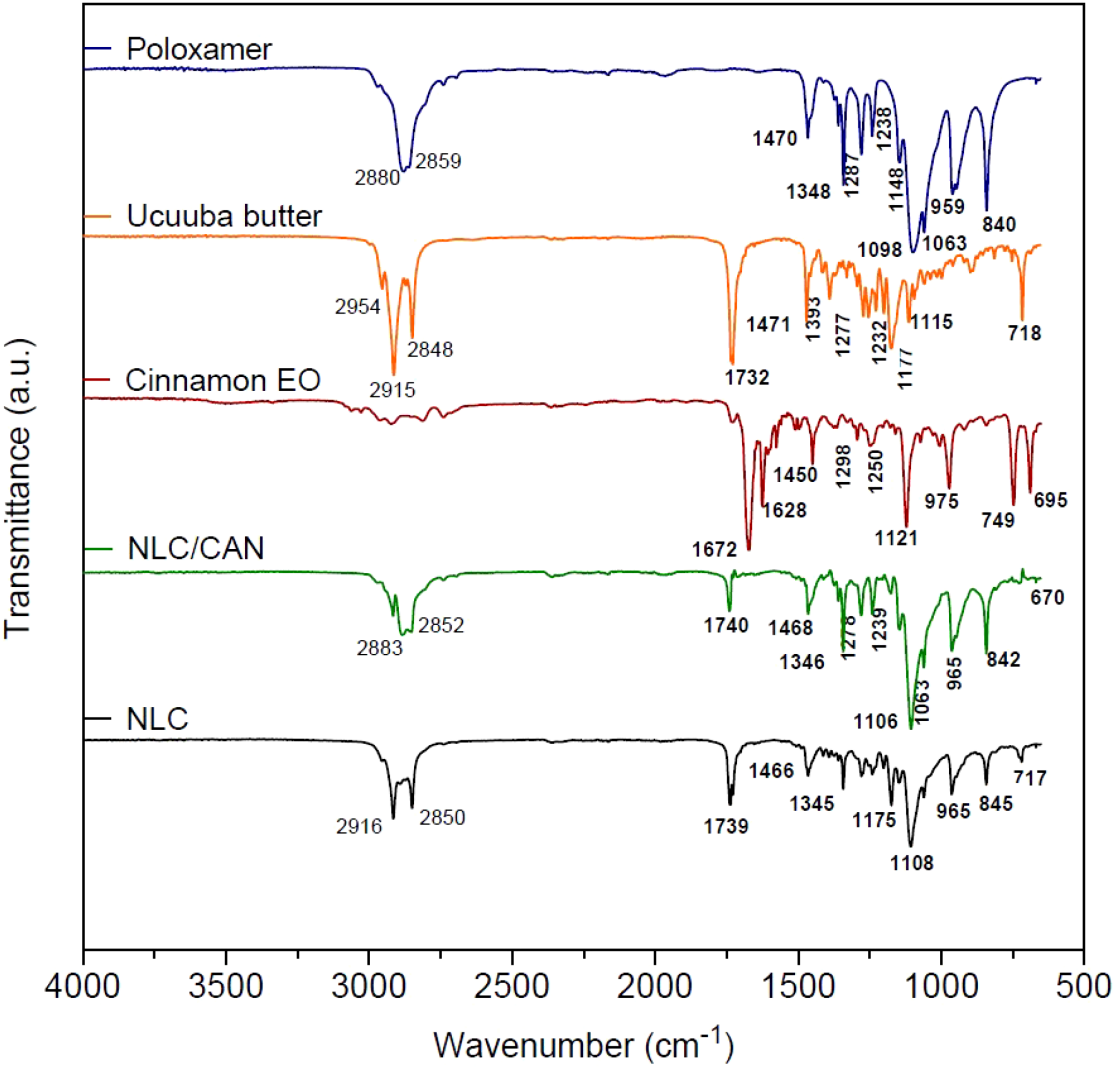
FTIR-ATR spectra in the region between 4000-500 cm^-1^ of NLC/CAN, NLC and its excipients.

Structural characterization was performed for NLC/CAN (F1), NLC control and its excipients. In FTIR-ATR analysis (**Figure 2**), the cinnamon EO spectrum revealed intense absorption bands at 1672 cm^-1^ (aldehyde C=O stretch), 1628 cm^-1^ (alkene C=C stretch) and 1450 cm^-1^ (C-C in the aromatic ring). In the vibrational spectrum of ucuuba butter, there are characteristic bands centered at 2915 cm^-1^ and a group of bands between 1393 and 1177 cm^-1^, corresponding to the –CH_2_ vibration. Strong bands centered at 1732 and 2848 cm^-1^ related to the vibration C=O and O-CH_2_, respectively, were also observed. The surfactant poloxamer showed typical bands to aliphatic chain ethers centered at 2880 and 1348 cm^-1^, associated with vibrations υO-CH_2_ and υO-C-O, respectively. In this same spectrum, it was evidenced characteristic bands of the symmetric deformation δCH_2_ at 1470 cm^-1^ and the axial stretching of CO (υCO) at 1098 cm^-1^.

FTIR-ATR spectra of NLC/CAN and NLC (control) revealed characteristic bands for these systems, resulting from the overlap of most of ucuuba butter and poloxamer bands, as expected. However, the NLC/CAN spectrum showed that the absorption band in the regions at 2852 - 2883 cm^-1^ became less intense when compared to the NLC spectrum in the region of 2850-2916 cm^-1^. Also, in the NLC/CAN spectrum, the absorption bands at 1672, 1628 and 1450 cm^-1^ observed in cinnamon EO were not detected.

**Figure 3:**
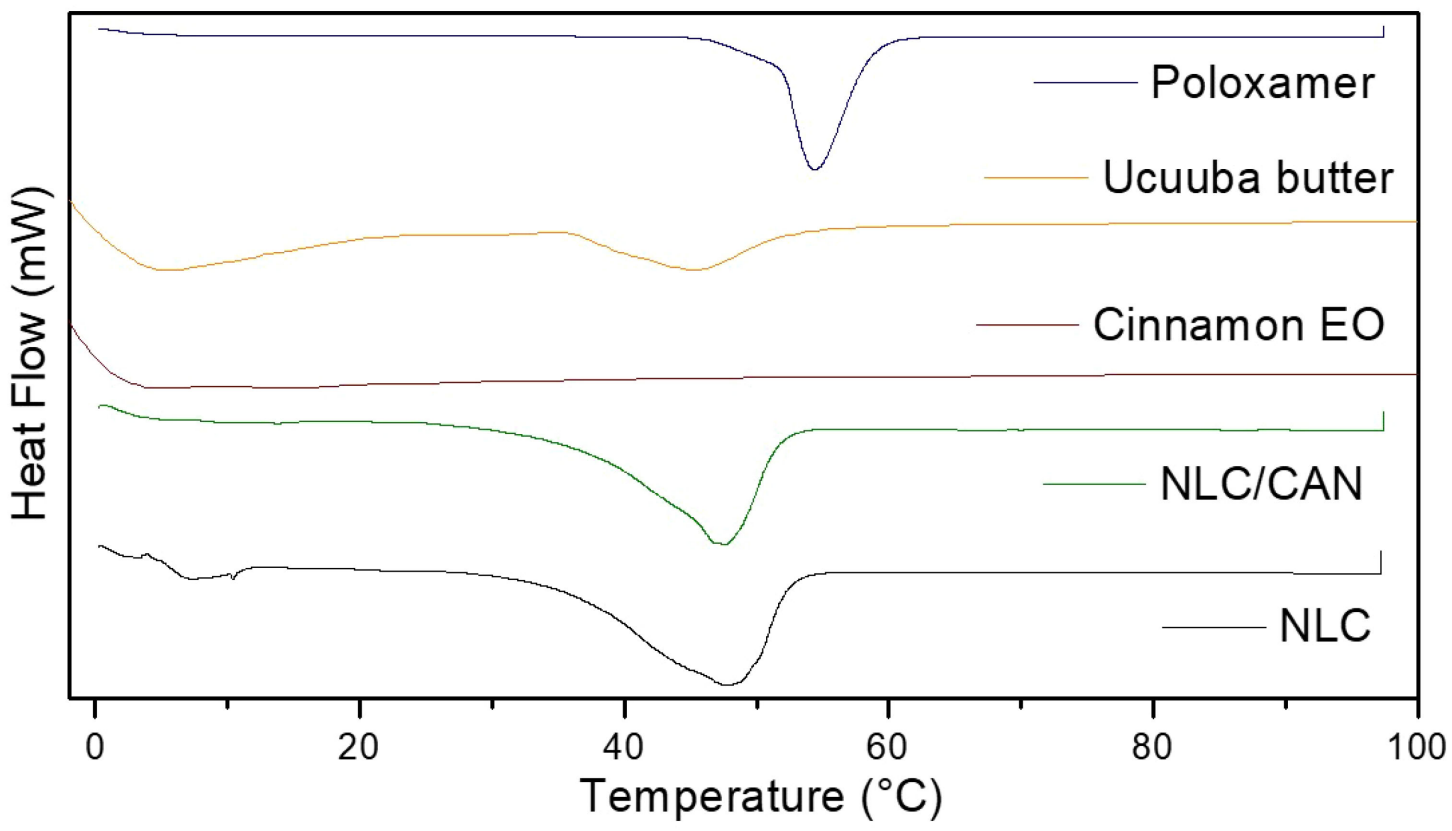
Thermogram obtained by DSC of NLC/CAN, NLC and its excipients.

Calorimetric analyses are necessary to demonstrate thermodynamic changes of formulations in relation to their excipients. **Figure 3** shows the thermograms of NLC control, NLC/CAN and all their excipients. DSC analysis showed that NLC/CAN presented a thermal profile compatible with its solid lipid matrix (ucuuba butter, 45.4°C), with the melting point of 47.1°C. Endothermic peaks corresponding to the melting point of poloxamer and NLC control was observed at 54.5 and 47.6°C, respectively.

**Figure 4:**
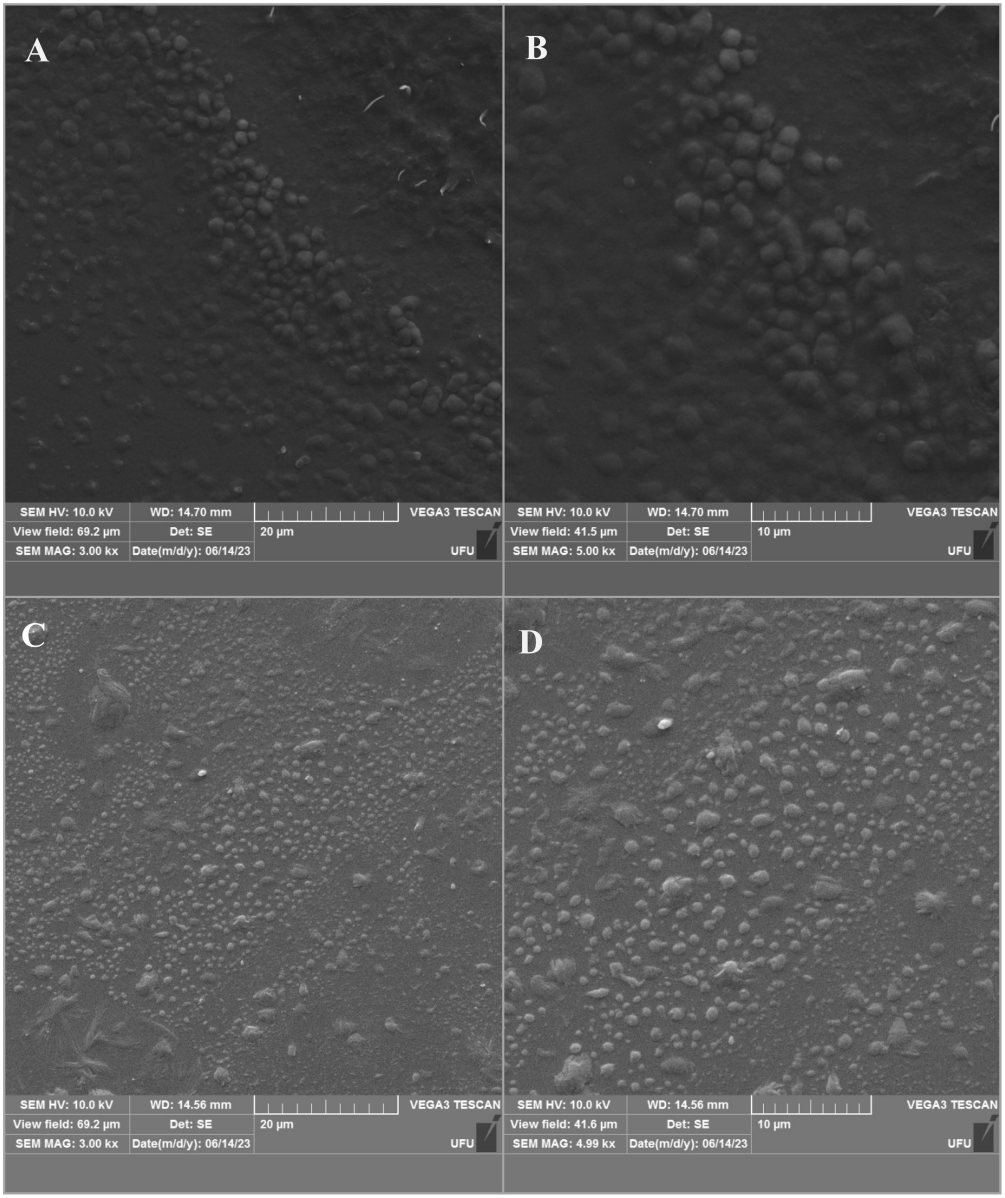
Scanning Electron Microscopy images of NLC/CAN **(A, B)** and NLC control **(C, D)**. The images are available in two different magnifications: 3000x (left) and 5000x (right).

FE-SEM images of NLC/CAN and NLC control showed that both formulations presented nanoparticles with spherical morphology and with well-defined contours, as expected for this type of system (**Figure 4**). In addition, the images revealed spherical monodisperse distributed nanoparticles, compatible with the morphology of NLC composed of synthetic and traditional lipids excipients (Ribeiro et al., 2016; Calixto et al., 2021; Castro et al., 2021; Moura et al., 2021).

### 3.5 Thermal cycling stability study

**Figure 5:**
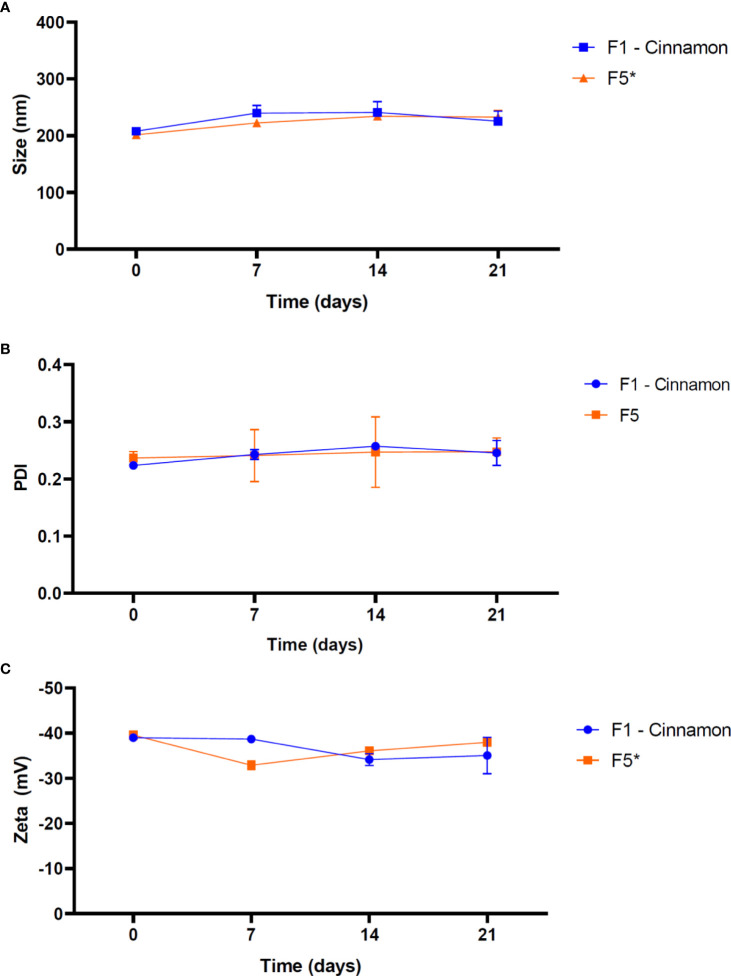
Thermal stability of NLC formulations in terms of size **(A)**, PDI **(B)** and Zeta potential **(C)**, at the end each temperature cycle. 1 cycle = 7 days of storage (4 days at 40°C followed by 3 days at 15°C). NOTE: One-way ANOVA/Tukey were used to analyze intragroup statistics; *p< 0.05. PDI: polydispersion index.

Thermal cycling stability study is useful to evaluate possible biophysical changes of nanoparticles under critical conditions of storage. **Figure 5** showed a thermal cycling stability study of the NLC/CAN and NLC (control) formulations.

Particle size of NLC/CAN exhibited no statistically significant changes over time, ranging from 208.00 nm to 241.20 nm at the end of 21 days. The PDI values obtained guaranteed that NLC/CAN presented a monodisperse size distribution even after storage under critical temperature conditions, which did not show statistically significant changes. ZP values ranged from - 34.13 to - 39.00 mV for NLC/CAN.

### 3.6 *In vivo* nanotoxicity test through chicken embryo model

**Figure 6:**
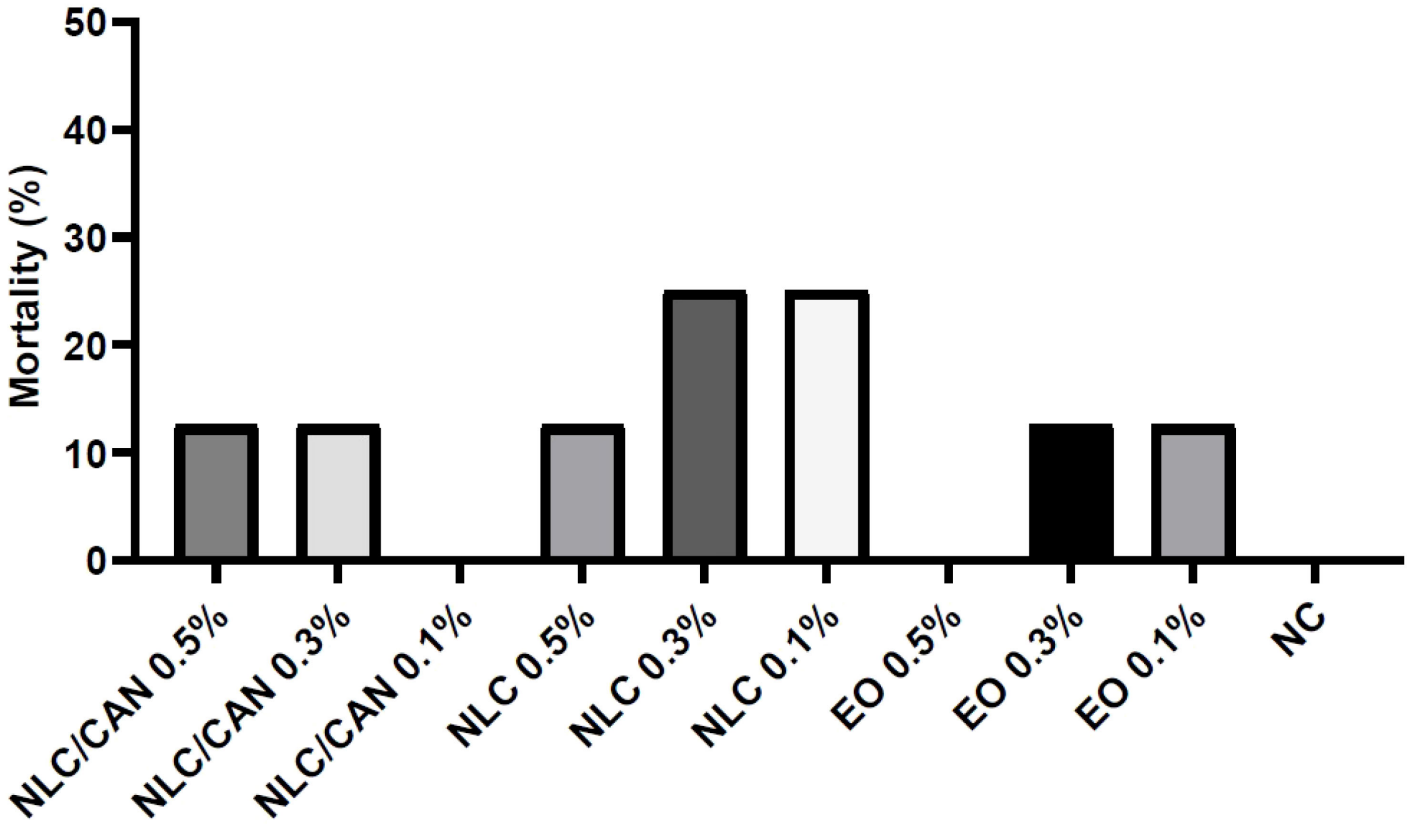
Mortality of chicken embryos treated with NLC/CAN, NLC and cinnamon EO emulsion (EO) at different concentrations.

**Figure 7:**
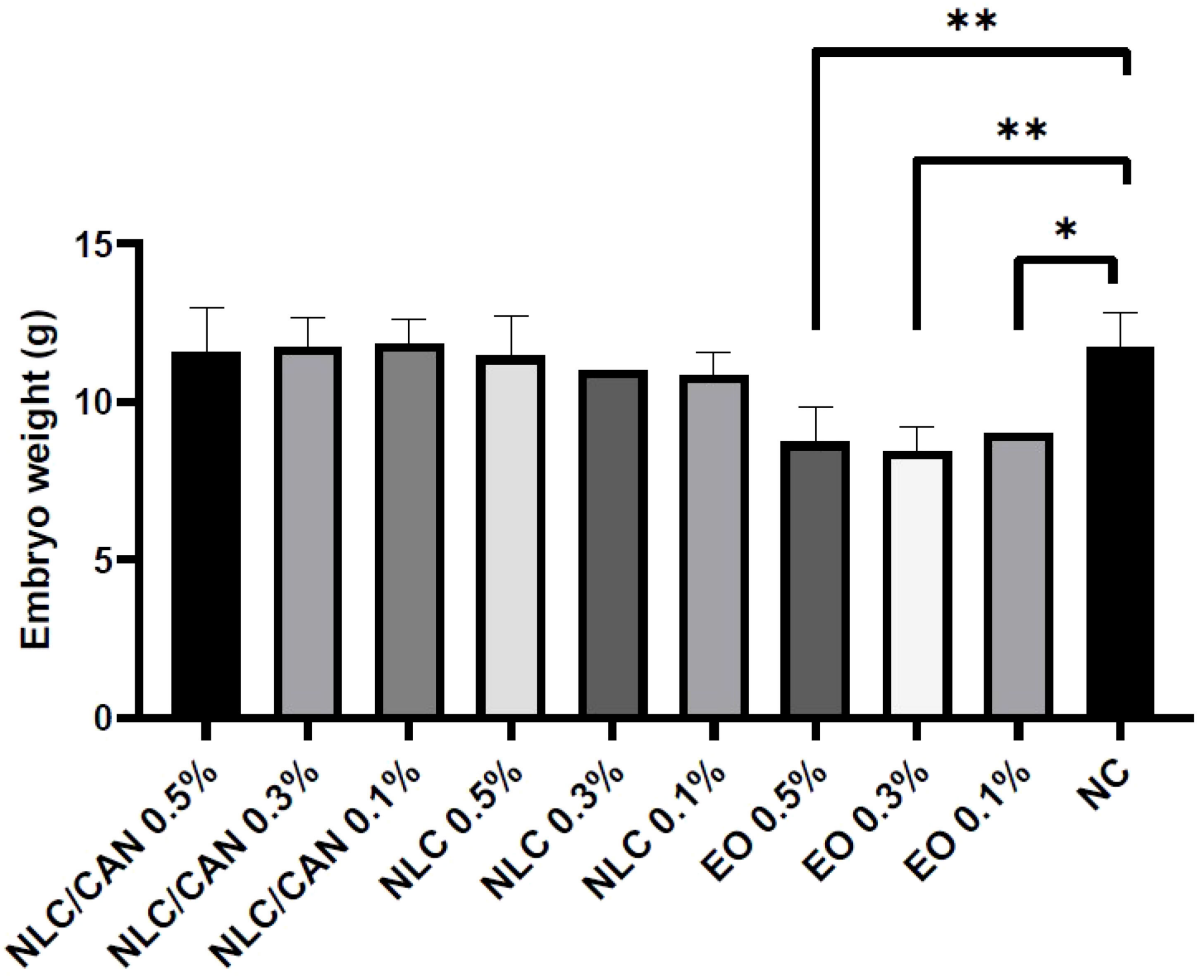
*In vivo* toxicity study in terms of chicken embryo weight under treatment with NLC/CAN, NLC and cinnamon EO emulsion (EO) at different concentrations. ** p< 0.01 and * p< 0.03.

**Figure 6** shows the mortality of embryos treated with negative control (NC = saline solution), NLC/CAN, NLC and cinnamon EO emulsified with poloxamer. There was no statistical difference between the treatments in comparison to the NC. Additionally, among alive embryos, there were no statistically significant macroscopic alterations compared to NC. However, regarding to the weight changes, the **Figure 7** demonstrates that there was a statistically significant difference (p< 0.05) in the weight of embryos treated with EO emulsion in comparison to the NC.

## 4 Discussion

Currently, there is a huge interest in the elucidation of the therapeutic properties of EO. There are several works that have described exciting results of EO uses in the cosmetic, agriculture, food and pharmaceutical fields (Castro et al., 2019; Guidotti-Takeuchi et al., 2022; Chukwuma et al., 2023).

The identification of bioactive compounds that represent an effective alternative to antibiotics is an important step in drug development in an attempt to minimize the bacterial resistance (Kaskatepe et al., 2016). In here, it was identified that cinnamon, oregano, peppermint and tea tree EO showed activity against the bacterial strains studied. However, the performance of oregano EO was strain-dependent in relation to *P. aeruginosa*, once a single strain showed resistance to this EO. Similar result was found for strains of *P. aeruginosa*, tested under the same conditions, showed different responses to the same EO (Coseriu et al., 2023), corroborating the difficulty to treat this type of infection. Other studies have also showed significant antibacterial properties for cinnamon EO, which inhibited the growth of *A. baumannii*, *P. aeruginosa* and *K. pneumoniae* (El-Kattan and Allam, 2021; Ganić et al., 2022; Coseriu et al., 2023). Furthermore, this EO exhibited higher antibiofilm activity against *K. pneumoniae*, followed by peppermint EO (Sarwar et al., 2022). Man et al. (2019) investigated the antibacterial activity of some EO against some Gram-positive and Gram-negative bacteria, including *K. pneumoniae* strains. It was also observed that oregano EO is a bioactive compound with promising antibiotic activity.

NLC formulations were monitored is terms of particle size (nm), polydispersion index (PDI) and Zeta potential (mV) in the long-term stability study in order to ensure the shelf time of such systems. This study is mandatory to ensure the quality control of the formulations. Regarding the particle size, there was not observed any tendency to increase in size as a function of time, which would be indicative of some physicochemical instability process, such as agglutination of nanoparticles. Moreover, regarding the size distribution profiles, a successful lipid nanocarriers should present homogeneous size distribution (monodisperse), with values as low as possible, to ensure long-term stability. Values ​​≤ 0.2 are usually accepted to describe a monodisperse distribution. Additionally, nanocarrier formulations with constant and narrow size distribution are correlated with better clinical results (Danaei et al., 2018). According to observed here, EO was responsible for reducing and making more uniform the particle size distribution of NLC when compared to the control formulation (without EO), elucidating its structural role in the system in addition to the already bioactivity claimed (Ribeiro et al., 2021). Furthermore, Zeta potential also brings information about the stability of colloidal aqueous dispersions (Souto et al., 2004). In general, good stability is achieved with Zeta potential values ​​above ±25 mV ensuring electrokinetic stabilization of the colloidal dispersion (Thatipamula et al., 2011). For sterically stabilized systems, as those use non-ionic as surfactants, the Zeta values ​​are more related to the sample composition, presenting slightly or moderately negative values, as observed here (Scioli Montoto et al., 2020)​​, indicating a desirable colloidal electrokinetic stability.

In addition, the thermal cycling study was carried out to bring some evidence regarding the control quality of NLC submitted to critical storage conditions. Such test provides information of the temperature and humidity conditions for a successful transport and distribution of NLC. In agreement with the long-term stability study. Such results strongly suggested that a cheap process to scale up, storage and distribution of such nanoformulations will be possible.

The *in vitro* antimicrobial activity of such formulations was determined by MIC. It was observed that the formulations that had cinnamon EO in their composition (F1, F6, F7 and F8) have showed activity against all the analysed strains/species and that the F1 formulation maintained *in vitro* antibacterial activity even after 1 year of storage at room temperature. Other work also revealed that the loaded cinnamon EO exhibited promising antibacterial activity and prevented both biofilm formation from *A. baumannii* strains, also exhibiting antibiofilm capacity (Ganić et al., 2022). Another work described that the strains of *P. aeruginosa* also showed susceptibility to cinnamon EO at lowest concentrations, (0.125% v/v). Such bactericidal effect of EO from the bark was higher than cinnamon leaf, probably due to the presence of Σ-cinnamaldehyde as the main component (85.3%). Furthermore, it was already shown that this EO induced rapid death of *P. aeruginosa* PAO-1, possibly due to the bacterial cell membrane rupture (Elcocks et al., 2020) and the synergistic action among its bioactive components, affecting gene expression and the activity of bacterial efflux pumps (Coseriu et al., 2023).

Then, it was selected NLC/CAN (F1) as the most effective nanoformulation. However, it is still necessary to continue investigating the efficacy of this nanosystem in more complex biological models, in order to obtain more robust results.

The F1 structural characterization through FTIR-ATR, DSC, FE-SEM methods, evidenced that the excipients were compatible with each other. In general, the F1 spectrum was based on the overlapped bands of ucuuba butter and poloxamer, their major components. However, in the NLC/CAN spectrum, there is strong evidence of interaction with EO, due to the absence of absorption bands in the regions at 1672, 1628 and 1450 cm^-1^ of cinnamon EO, suggested an excellent miscibility between ucuuba butter and EO. Furthermore, NLC have showed thermal stability up to 100°C, without any thermal event related to the degradation of fatty acids, as required for this type of system. The structural and thermal analyses have also confirmed that the EO is part of the structure of the NLC, providing lower viscosity and crystallinity to the lipid matrix (Ribeiro et al., 2021). Such achieved corroborated the complex supramolecular arrangement of NLC in comparison with SLN (composed of only solid lipid in the structural matrix). The blend of lipids results in a more amorphous form with structural imperfections resulting in more stable and efficient formulations (Souto et al., 2004).

Finally, the safety of NLC/CAN formulation was ensured through the *in vivo* nanotoxicity test on CE model. All the nanoformulations and concentrations tested were safe, once they did not show any statistically significant differences in comparison to the NC, in all the parameters analysed. However, the CE weight changes treated with cinnamon EO emulsion was statistically significant different (p< 0.05) compared to the NC, confirming the protective effect of loading EO by NLC. It is worth mentioning that emulsion was the first colloidal delivery systems already reported, and it is widely known that system is unstable, being not applicable as treatment candidate or nanosanitizer (Araújo et al., 2019). This approach had also determined the higher safe dose on embryos for further efficacy tests. Moreover, it was observed that NLC/CAN, composed of cinnamon EO, ucuuba butter and poloxamer was safe up to 50 mg/mL of cinnamon EO loaded by NLC, much higher than the effective concentration value given by MIC, which was around 1.2 mg/mL.

The benefits of nanostructured drugs can be realized mainly for cancer treatments, with some nanocolloidal systems applied as gold standard treatments, as Doxil^®^ (Ben Venue Laboratories, Inc Bedford, OH) and Myocet^®^ (GP-Pharm, Barcelona, ​​Spain). In the commercial antimicrobials nanoparticle-based drugs, 3 lipid-based formulations of amphotericin B are commonly used for the systemic treatment of fungal infections: Ambisome^®^ (Gilead Sciences, Inc, San Dimas, CA), Amphotec^®^ (Ben Venue Laboratories, Inc, Bedford, OH) and Abelcet^®^ (Sigma-Tau PharmaSource, Inc, Indianapolis, IN) (Adler-Moore and Proffitt, 2002; Yeh et al., 2011). Despite the excellent biophysical and biological activity of nanoformulations, there are a lot of challenges to be overcome until they reach the market. This is still necessary considering the particularities on nanostructured system in the regulatory issues, as well as in the biological assays planning, in order to decrease the time of approval and provided standardized methods to specifically evaluated the nanotoxicity in the biological tissues and environment.

The data found in here strongly suggest that NLC/CAN formulation remained stable up to 12 months, exhibiting excellent properties in terms of particle size, homogeneity and surface charge at the end of experiment. So, the NLC/CAN is a promising system, being economically viable to be used as a surface disinfectant or antiseptic of hospitals. Besides, the same system has structural properties that allows its administration on parenteral and topical (skin and mucosa) routes, aiming to be a safe and more efficient antibiotic treatment against multidrug resistant species.

## 5 Conclusions

NLC formulations that encapsulated EO demonstrated excellent long-term stability (1 year, at 25°C). Such system was effective against *Acinetobacter baumannii*, *Klebsiella pneumoniae* and *Pseudomonas aeruginosa* isolated from humans. The structural characterization confirmed a supramolecular organization compatible with NLC formulations and thermal stability of the systems up to 100°C, making them suitable for use in biomedical applications. The evaluation of nanotoxicity in embryos showed that the NLC/CAN formulation was safe under all conditions tested. Furthermore, it was observed that the safe concentration was higher than the effective one. Based on these promising results, the NLC/CAN will be further submitted *in vivo* efficacy studies on chicken embryo and mammal model, in order to find an effective, safe and low-cost therapeutic option against infections by multidrug-resistant bacteria.

## Data availability statement

The original contributions presented in the study are included in the article/Supplementary Material. Further inquiries can be directed to the corresponding author.

## Ethics statement

The manuscript presents research on animals that do not require ethical approval for their study.

## Author contributions

Ed: Conceptualization, Data curation, Formal Analysis, Investigation, Methodology, Visualization, Writing – original draft, Writing – review & editing. LB: Funding acquisition, Project administration, Resources, Supervision, Writing – review & editing. BF: Methodology, Formal analysis, Data curation, Funding acquisition, Project administration, Resources, Supervision, Writing – review & editing. RR: Writing – review & editing. SS: Investigation, Writing – review & editing. HP: Investigation, Writing – review & editing. Fd: Investigation, Writing – review & editing. LR: Conceptualization, Data curation, Formal Analysis, Funding acquisition, Investigation, Methodology, Project administration, Resources, Supervision, Visualization, Writing – review & editing.
